# Sodium Alginate/Cuprous Oxide Composite Materials with Antibacterial Properties: A Preliminary Study Revealing the Counteracting Effects of Oligosaccharides in the Matrix

**DOI:** 10.3390/foods14101666

**Published:** 2025-05-08

**Authors:** Reeba Thomas, Fengyi Wang, Wipa Suginta, Chien-Yi Chang, Fengwei Xie

**Affiliations:** 1 School of Biomolecular Science and Engineering, Vidyasirimedhi Institute of Science and Technology, Rayong 21210, Thailand; reebathomas94@gmail.com (R.T.); wipa.s@vistec.ac.th (W.S.); 2 School of Engineering, Newcastle University, Newcastle upon Tyne NE1 7RU, UK; 3 School of Dental Sciences, Faculty of Medical Sciences, Newcastle University, Newcastle upon Tyne NE2 4BW, UK; f.wang2@newcastle.ac.uk (F.W.); chienyi.chang@newcastle.ac.uk (C.-Y.C.); 4 Department of Chemical Engineering, University of Bath, Bath BA2 7AY, UK; 5 Nottingham Ningbo China Beacons of Excellence Research and Innovation Institute, University of Nottingham Ningbo China, 211 Xingguang Road, Ningbo 315048, China

**Keywords:** chitin oligosaccharides, chitosan oligosaccharides, sodium alginate hydrogels, cuprous oxide, antimicrobial properties, polysaccharide composites

## Abstract

The integration of biopolymers with antimicrobial inorganic materials has emerged as a promising strategy for developing eco-friendly and biocompatible functional materials for food packaging and biomedical applications. However, the impact of biopolymer matrix composition on the antimicrobial efficacy of inorganic fillers remains underexplored. This study addresses this critical gap by investigating the effects of chitin or chitosan oligosaccharides (NACOS or COS) on the antimicrobial properties of sodium alginate (SA)/cuprous oxide (Cu_2_O) composite gels. The composite gels were synthesized through a physical blending of the components, followed by calcium-induced crosslinking of SA. Characterization using UV-vis, FTIR, and EDX confirmed the successful incorporation of Cu_2_O, while a SEM analysis revealed its uniform dispersion. Antibacterial assays demonstrated that SA-Cu_2_O exhibited the highest inhibition rates, with a 67.4 ± 11.9% growth suppression of *Staphylococcus aureus* (MRSA), 33.7 ± 5.1% against *Escherichia coli*, and 39.1 ± 14.8% against *Pseudomonas aeruginosa*. However, incorporating NACOS and COS reduced inhibition, as oligosaccharides served as bacterial carbon sources. Swelling and contact angle measurements indicate that antimicrobial effectiveness was independent of surface hydrophilicity. These findings underscore the importance of rational composite design to balance bioactivity and material stability for antimicrobial applications.

## 1. Introduction

Bacterial contamination poses a significant challenge in food safety, leading to product spoilage, foodborne illness, and economic losses in the food industry. Pathogenic bacteria such as *Escherichia coli*, *Pseudomonas aeruginosa*, and *Staphylococcus aureus* are among the most prevalent foodborne contaminants, contributing to severe health risks and regulatory concerns [1,2]. The emergence of antimicrobial-resistant strains further exacerbates these risks, necessitating the development of effective antimicrobial packaging materials to enhance food preservation and safety [3].

Biopolymers, which are naturally occurring macromolecules, have attracted considerable interest owing to their applications in diverse fields, such as biomedicine, pharmaceuticals, food preservation, agriculture, chemical processes, energy, and environmental remediation [4,5,6,7,8,9,10,11]. Among these, polysaccharides such as cellulose, chitin/chitosan, sodium alginate (SA), and starch, as well as proteins such as collagen/gelatin and whey protein, have been extensively investigated for their utility in hydrogel/aerogel development. These biopolymer-based gels feature a three-dimensional crosslinked network capable of swelling in aqueous environments while retaining structural integrity, rendering them highly suitable for a broad range of applications [12]. Particularly, SA can form a robust 3D crosslinked network through ionic linkages mediated by multivalent cations, such as Ca^2^⁺. [9,10]. When combined with antimicrobial agents, SA-based materials can serve as active packaging solutions that extend shelf life and inhibit microbial growth on food surfaces [9,10,13]. Recent advancements in incorporating metal inorganic compounds, such as cuprous oxide (Cu_2_O), into SA matrices have demonstrated enhanced antibacterial efficacy [14]. Cu_2_O is known for its broad-spectrum antibacterial activity, primarily through mechanisms involving bacterial membrane disruption, oxidative stress induction, and metabolic interference [15].

Chitin and its derivatives, including chitin oligosaccharides (NACOS) and chitosan oligosaccharides (COS), have been widely explored for their antimicrobial and functional properties in therapeutic applications [16]. These oligosaccharides exhibit intrinsic antimicrobial activity, promote wound healing, and enhance the physicochemical properties of biopolymer films [17,18]. Recent studies [19] have demonstrated that SA/COS gel beads exhibit antibacterial effects against *S. aureus* and *E. coli*, with their physicochemical properties being influenced by the calcium ion concentration and molecular weight variations. The study highlighted that incorporating COS led to improved antibacterial effects and denser cross-sectional structures, making these materials promising candidates for food industry applications. However, recent studies have highlighted the dual role of these oligosaccharides in antimicrobial systems. While COS has been reported to disrupt bacterial membranes and inhibit biofilm formation [20], its low molecular mass and high solubility may allow bacterial metabolism, potentially reducing the antimicrobial efficacy of composite materials [21].

Despite advancements in biopolymer–inorganic antimicrobial composites, the interplay between oligosaccharides and inorganic metal in sodium alginate matrices remains underexplored. Most existing studies focus on the individual antimicrobial effects of Cu_2_O or oligosaccharides but do not address their combined impact in composite systems.

This study investigates composite materials comprising SA and Cu_2_O particles, further added with COS or NACOS, to evaluate their antibacterial properties. The primary objective is to elucidate how varying polysaccharide matrices influence the antimicrobial efficacy of Cu_2_O. These composite hydrogels were designed to harness the biocompatibility and gel-forming ability of SA, which serves both as a dispersant and carrier of Cu_2_O particles, alongside the bioactive functionalities of COS and NACOS. Antimicrobial efficacy was assessed against Gram-positive (*S. aureus*) and Gram-negative (*E. coli* and *P. aeruginosa*) bacterial strains. A key focus was to determine whether the polysaccharide/oligosaccharide components in the matrix could act as carbon sources for bacterial growth, potentially counteracting the antimicrobial effects of Cu_2_O particles, or provide additional antimicrobial effects. This study advances our understanding of the role of polysaccharides and their derivatives in antibacterial activity, offering valuable insights into the rational design of biopolymer-based materials with antibacterial properties in applications such as food packaging and biomedicine. The findings contribute to the growing body of knowledge on sustainable antimicrobial materials by addressing the potential trade-offs between the bioactive benefits and nutrient-like behavior of oligosaccharides. This research aims to optimize the formulation of biopolymer-based composites for targeted applications.

## 2. Materials and Methods

### 2.1. Materials and Chemicals

The materials used in this work include sodium alginate (CP, 200 ± 20 mpa.s, Shanghai Macklin Biochemical Co., Ltd., Shanghai, China), *N*-acetyl chitooligosaccharides (NACOS, or chitin oligosaccharides, Tokyo Chemical Industry Co., Ltd., Oxford, UK), chitosan oligosaccharides (COSₙ = 2–6, Tokyo Chemical Industry Co., Ltd., Oxford, UK), copper(I) oxide power (Cu_2_O, Cat. No. 197425000, Thermo Fisher Scientific, Cramlington, UK; particle size up to a few microns, as characterized by scanning electron microscopy (SEM)), calcium chloride (CaCl_2_, 110.99, Sigma Aldrich (Merck, Gillingham, UK), ethanol absolute (analytical reagent grade, Fisher Scientific, Cramlington, UK), tryptic soy broth (Thermo Fischer Scientific, Cramlington, UK), and sodium hydroxide (Thermo Fisher Scientific, Cramlington, UK).

### 2.2. Preparation of SA-Based Hydrogel Beads

For preparing the SA-NACOS-Cu_2_O beads, 1 g of SA, 1 g of NACOS, and 2 g of Cu_2_O were mixed in 100 mL of DI water using a stirrer until a homogeneous solution was achieved. The resulting solution was then dropped into 0.1 M calcium chloride (CaCl_2_) using a 10 mL syringe to form hydrogel beads. These beads were soaked in CaCl_2_ for 18 h and subsequently washed in DI water. Additionally, the beads were rinsed several times with ethanol (100%) before being dried in a supercritical drier (Polaron, E3000 Critical Point Drying Apparatus, Sussex, UK), with the temperature and pressure set to be 34 °C and 1100 psi (7.58 MPa), respectively. After drying, the samples were stored in desiccators. The same procedure was followed to prepare SA-NACOS beads without Cu_2_O.

To prepare the SA-COS and SA-COS-Cu_2_O beads, 1 g of COS was dissolved in 10 mL of 1 M sodium hydroxide (Thermo Fisher Scientific, UK), while 1 g of SA was separately dissolved in 90 mL of deionized water, making a final volume of 100 mL. The two solutions were combined and mixed thoroughly until a homogeneous solution was obtained. The subsequent bead formation, crosslinking, washing, and drying steps followed the same procedure as described above for the SA-NACOS-Cu_2_O beads.

Table 1 presents the details of the different samples involved in this work.

### 2.3. Material Characterization

#### 2.3.1. UV-Vis Spectroscopy

For UV-vis spectroscopy analysis, 5 mg of the sample were immersed in 5 mL of deionized water in a clean glass vial and allowed to equilibrate for 24 h at room temperature. The presence of Cu_2_O in the composites was confirmed using a Jenway Model 7315 UV/visible single-beam spectrophotometer (Bibby Scientific Limited, Stone, UK) with the measurements conducted across a wavelength range of 190–800 nm. The instrument was pre-warmed and baseline-corrected using a blank (deionized water) in a clean quartz cuvette. The hydrogel solution was then carefully transferred into a cuvette using a pipette. The spectra were analyzed to identify the characteristic absorption peaks and plotted using GraphPad Prism software v5.01.

#### 2.3.2. Morphological Analysis

Scanning electron microscopy (SEM) and energy-dispersive X-ray (EDX) spectroscopy were performed using an Oxford Instruments AZtec EDX system (Oxford Instruments Nanoanalysis Buckinghamshire, UK) equipped with an X-act thin-window detector, featuring an energy resolution of 129 eV at the Mn Kα line (5.9 keV). The system was operated using AZtecLive software (v6.1) for real-time elemental mapping and spectral acquisition. Prior to analysis, the samples were carbon-coated to facilitate accurate chemical composition assessment.

#### 2.3.3. Fourier-Transform Infrared (FTIR) Spectroscopy

The chemical functional groups in the composite were characterized using attenuated total reflectance Fourier-transform infrared (ATR-FTIR) spectroscopy, performed with a Cary 630 spectrometer (Agilent Technologies, Santa Clara, CA, USA). Measurements were conducted over a spectral range from 400 to 4000 cm^−1^ with a resolution of 4 cm^−1^.

#### 2.3.4. Swelling Assay

The water absorption capacity of the developed beads was evaluated following previously established methods [22]. Briefly, 0.1 g of beads were weighed and immersed in a petri dish containing 10 mL of deionized water. At predetermined time intervals, the beads were removed, excess water was blotted using tissue paper, and their weight was recorded.

The swelling ratio of the beads was calculated using Equation (1):

(1) $$Swelling\;ratio\;(\%)=\frac{W_{t}-W_{0}}{W_{0}}\times 100$$

where *W*_0_ represents the initial weight of the beads, and *W*_t_ denotes the weight of the beads at time *t*.

#### 2.3.5. Water Contact Angle (WCA) Analysis

To evaluate surface wettability, a homogeneous solution of each composite formulation was cast into a Petri dish and dried at 50 °C in an oven. After drying, the composite film was sectioned into smaller pieces and mounted on the stage of an Ossila contact angle goniometer (Ossila Ltd., Sheffield, UK). A 16 μL water droplet was deposited onto the film surface, and the contact angle was measured using the instrument’s integrated software (v4.1.5).

#### 2.3.6. Antibacterial Assay

The antibacterial activity was evaluated using *E. coli* DH5α, *P. aeruginosa* PAO1 Nottingham subline [23], and methicillin-resistant *Staphylococcus aureus* (MRSA) USA300. *E. coli* and *P. aeruginosa* were cultured in Luria broth, while MRSA was grown in tryptic soy broth at 37 °C with shaking at 200 rpm overnight. Polymer composites were sterilized by autoclaving at 121 °C with a pressure of 15 pounds per square inch for 15 min. Sterilized polymer beads (5 mg·mL^−1^) were introduced into the media inoculated with diluted MRSA, *P. aeruginosa*, and *E. coli* (optical density at 600 nm wavelength, OD_600_ = 0.01) from overnight cultures. The cultures were incubated for 18 h at 37 °C with shaking at 200 rpm. Negative controls consisted of cultures without polymer addition. Each experiment was performed in triplicate (*n* = 3) to ensure statistical reliability. Bacterial growth was monitored by measuring the optical density at 600 nm (OD_600_). Growth inhibition was calculated and plotted using GraphPad Prism software v5.01. A two-way analysis of variance (ANNOVA) was conducted to evaluate which composite was inhibited more and the bacterial strain on inhibition. Statistical significance was set at *p <* 0.05.

The percentage of inhibition was determined using Equation (2):

(2) $$\%\;inhibition=\frac{1-Abs\;of\;sample}{Abs\;of\;control}\times 100$$

#### 2.3.7. Microbial Activity Under Different Carbon Sources

To investigate the influence of SA, NACOS, COS, and *N*,*N*-diacetylchitobiose [(GlcNAc)_2_] on MRSA growth, bacteria were inoculated into minimal media (M9) devoid of these substrates. M9 media was prepared using M9 salts (5×), magnesium sulfate (1 M), calcium chloride (1 M), and water to make a final volume of 100 mL. Magnesium sulfate was filter sterilized, while the remaining components were autoclaved before use. Bacterial growth was monitored by measuring the optical density at 600 nm (OD_600_), with glucose serving as the control.

## 3. Result and Discussion

### 3.1. Morphology and Composition Characterization

**Figure 1:**
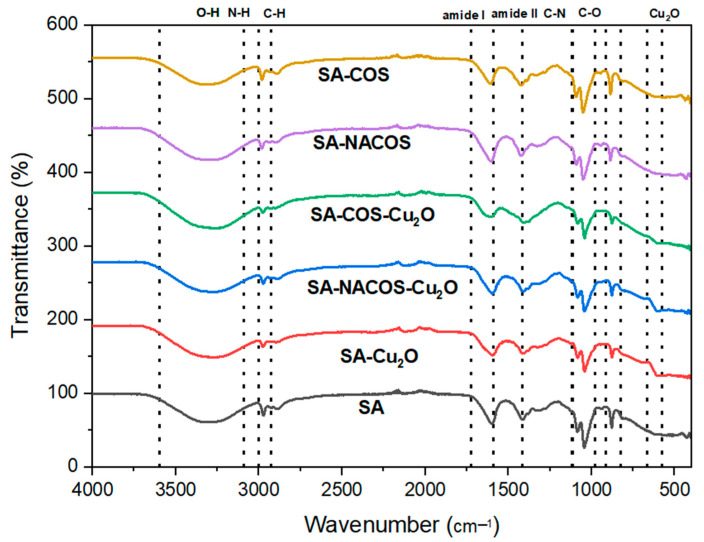
FTIR spectrum of sodium alginate (SA), sodium alginate-cuprous oxide (SA-Cu_2_O), sodium alginate-chitin oligosaccharides-cuprous oxide (SA-NACOS-Cu_2_O), sodium alginate-chitosan oligosaccharides-cuprous oxide (SA-COS-Cu_2_O), sodium alginate–chitin oligosaccharides (SA-NACOS), and sodium alginate–chitosan oligosaccharides (SA-COS).

Figure 1 presents the FTIR spectra of the composites. The SA spectrum exhibits a broad characteristic absorption band in the range of 3600-3000 cm^−1^, corresponding to O–H stretching, indicative of the polysaccharide’s extensive hydrogen bonding. The peaks at 3000-2800 cm^−1^ correspond to C–H stretching vibrations, while the asymmetric and symmetric stretching of carboxylate (COO^−^) groups are represented by the peaks at approximately 1600 cm^−1^ and 1400 cm^−1^, respectively, confirming the presence of alginate’s carboxyl functionalities.

Upon the introduction of chitosan (SA-COS) and *N*-acetyl glucosamine (SA-NACOS), additional amide-related bands emerge. The amide I band, attributed to C=O stretching, is observed around 1650 cm^−1^, while the amide II band, associated with N–H bending, appears at ~1550 cm^−1^. In *N*-acetyl glucosamine composites, an amide III band at approximately 1312 cm^−1^ indicates C–N stretching, further distinguishing these materials from pure SA. These spectral shifts suggest interactions between alginate and the amine-functionalized oligosaccharides, potentially through hydrogen bonding and electrostatic interactions, modifying the composite structure.

The incorporation of Cu_2_O in SA-Cu_2_O, SA-COS-Cu_2_O, and SA-NACOS-Cu_2_O results in additional spectral features, particularly in the range of 550–670 cm^−1^, corresponding to Cu–O vibrations. Additionally, Cu_2_O-containing samples exhibit changes in the intensities and positions of hydroxyl and amide peaks, suggesting interactions between Cu_2_O and the polymeric components.

**Figure 2:**
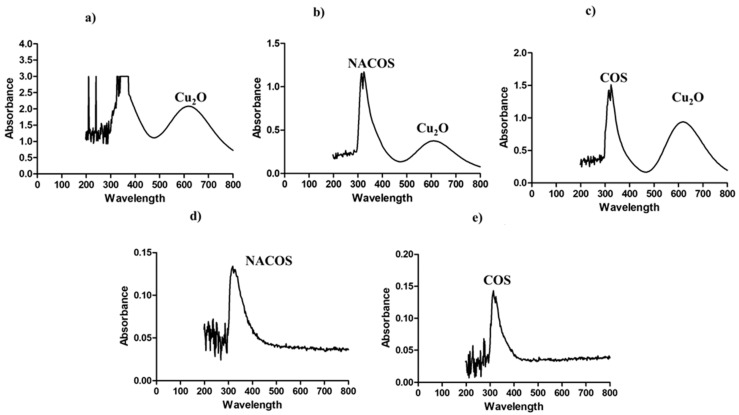
UV-visible spectra of (**a**) SA-Cu_2_O, (**b**) SA-NACOS-Cu_2_O, (**c**) SA-COS-Cu_2_O, (**d**) SA-NACOS, and (**e**) SA-COS.

Figure 2 presents the UV-vis spectra of the composites, revealing a Cu_2_O peak at a wavelength of 600 nm, while peaks corresponding to NACOS and COS were observed near 300 nm. These results demonstrate the successful incorporation of Cu_2_O particles into the SA matrix.

**Figure 3:**
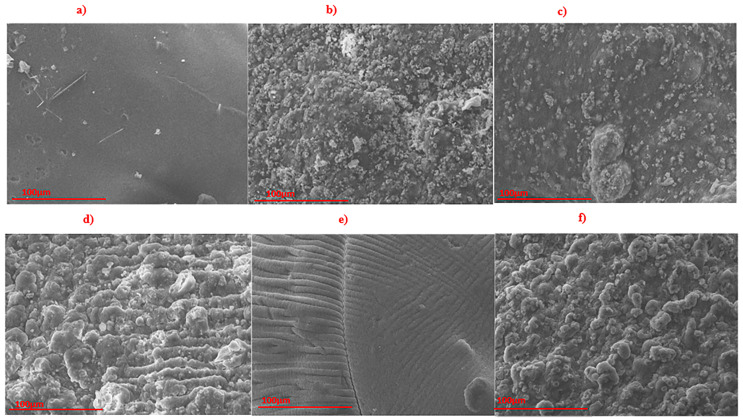
SEM images of (**a**) SA, (**b**) SA-Cu_2_O, (**c**) SA-NACOS-Cu_2_O, (**d**) SA-COS-Cu_2_O, (**e**) SA-NACOS, and (**f**) SA-COS.

The surface morphology of the composite beads, analyzed using SEM, is depicted in Figure 3. The pure sodium alginate (SA) sample exhibited an exceptionally smooth and dense surface with minimal discernible features. The incorporation of NACOS into SA did not significantly alter the surface morphology, suggesting the formation of a relatively homogeneous phase structure between NACOS and SA. In contrast, the SA-COS sample displayed a rough, granular surface characterized by a loose microstructure.

Theoretically, the interaction between SA and COS is expected to be stronger than that between SA and NACOS. This is attributed to the presence of amine groups in COS, which carry a positive charge under mild acidic conditions, while alginate, with its carboxyl groups, is negatively charged. Consequently, electrostatic interactions and hydrogen bonding can occur between SA and COS. In contrast, NACOS, due to its acetyl groups, does not facilitate such electrostatic interactions with SA. Additionally, the polarity of the acetyl groups is likely to be lower than that of the amine groups, resulting in weaker hydrogen bonding between SA and NACOS compared to that between SA and COS. Therefore, the compatibility between SA and COS is expected to be greater than that between SA and NACOS. However, it is important to note that the granules observed on the surface of the SA-COS film may represent coacervates formed within the SA matrix due to the electrostatic interactions between SA and COS, leading to the coarse and less-dense microstructure of SA-COS observed under SEM.

The incorporation of Cu_2_O into SA or SA-NACOS yielded a morphology characterized by uniformly dispersed small particles, likely corresponding to Cu_2_O particles. These observations indicate that the SA matrix served as an effective dispersant for Cu_2_O particles, potentially mediated by ionic interactions between Cu_2_O and SA. Furthermore, the SA-COS-Cu_2_O composite displayed a surface morphology similar in coarseness to that of the SA-COS sample.

**Figure 4:**
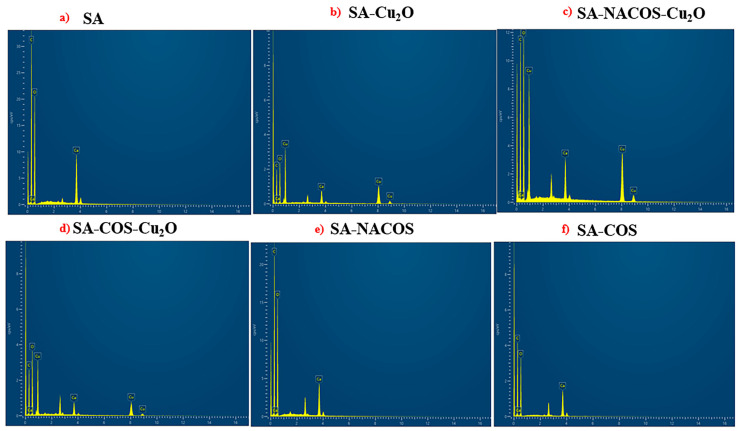
EDX spectra of (**a**) SA, (**b**) SA-Cu_2_O, (**c**) SA-NACOS-Cu_2_O, (**d**) SA-COS-Cu_2_O, (**e**) SA-NACOS, and (**f**) SA-COS. The SA spectrum (**a**) displays peaks for carbon (C), oxygen (O), and calcium (Ca). In SA-Cu_2_O (**b**), additional peaks corresponding to copper (Cu) confirming the incorporation of Cu_2_O. Spectra of SA-NACOS-Cu_2_O (**c**) and SA-COS-Cu_2_O (**d**) reveal the presence of C, O, Ca, and Cu, indicating successful integration of NACOS or COS with Cu_2_O. The spectra of SA-NACOS (**e**) and SA-COS (**f**) show peaks for C, O, and Ca, confirming the presence of the respective oligosaccharides without copper.

The EDX spectra presented in Figure 4 confirmed the presence of carbon, oxygen, copper, and calcium. The addition of Cu_2_O into the SA-NACOS and SA-COS results in a slight reduction in copper ions. This is probably due to the incorporation into the structure, thereby reducing the availability in the system.

### 3.2. Swelling Behavior

**Figure 5:**
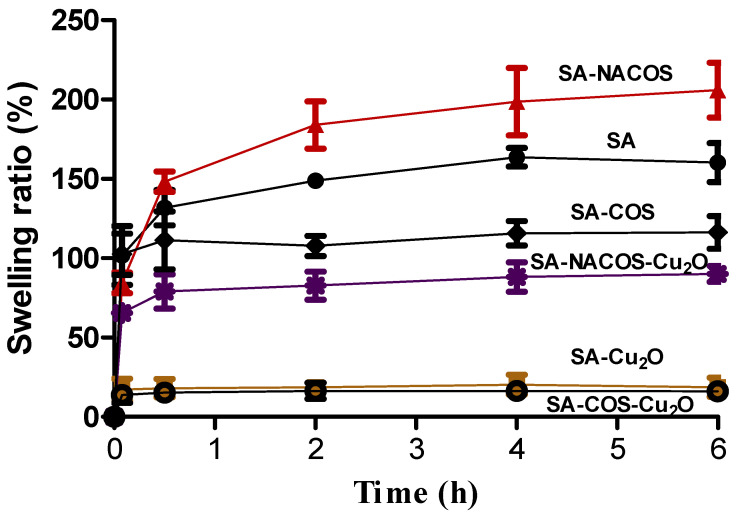
Swelling ratio (%) for the sodium alginate (SA) film and the different composite (SA-Cu_2_O, SA-NACOS, SA-NACOS-Cu_2_O, SA-COS, and SA-COS-Cu_2_O) films.

Swelling quantifies the extent to which a material expands or increases in volume upon exposure to specific liquid environments. Investigating water swelling behavior is critical for assessing a material’s hygroscopicity and predicting its stability or water absorption capacity in water or under high-moisture conditions. The swelling properties of the developed composites, namely SA-Cu_2_O, SA-NACOS, SA-NACOS-Cu_2_O, SA-COS, and SA-COS-Cu_2_O in the form of dried beads, were evaluated in water at intervals of 5 min, 30 min, 2 h, 4 h, and 6 h (see Figure 5).

Upon water exposure, the swelling order of the composites was as follows: SA-NACOS > SA > SA-COS > SA-NACOS-Cu_2_O > SA-Cu_2_O > SA-COS-Cu_2_O (see Table 2). Among the composites, SA-NACOS exhibited the highest water absorption capacity, reaching approximately 203.9 ± 17.3% at 6 h upon achieving swelling equilibrium, which is attributed to its pronounced hydrophilic nature. This was followed by the pure SA composite (160.4 ± 12.2% at 6 h) and the SA-COS composite (116.1 ± 10.3% at 6 h). These findings indicate that NACOS possesses greater hydrophilic properties than SA, while the interaction between SA and COS rendered the SA-COS beads less prone to swelling in water.

The inclusion of Cu_2_O into the SA, SA-COS, and SA-NACOS samples (i.e., SA-Cu_2_O, SA-COS-Cu_2_O, and SA-NACOS-Cu_2_O) significantly reduced swelling, yielding capacities of 18.6 ±6.0%, 16.1 ±3.9%, and 90.1 ± 4.9% at 6 h, respectively. This reduction is attributed to the hydrophobic nature of Cu_2_O, which diminishes the overall hygroscopicity of the SA-based composites. The decreased water swelling in the Cu_2_O-contained SA samples is particularly advantageous for applications such as food packaging and marine coatings, where materials must perform underwater or in highly moistened conditions. The swelling behavior of these composites highlights the balance between the hydrophilic properties of biopolymers (SA, NACOS, and COS) and the hydrophobic influence of Cu_2_O particles.

### 3.3. Surface Hydrophilicity

Contact angle is a fundamental parameter for evaluating the wettability of a solid surface by a liquid, defined as the angle between the tangent line to the liquid’s surface and the solid surface at the point of contact. A WCA greater than 90° signified hydrophobicity, while a value below 90° indicates hydrophilicity [24].

**Figure 6:**
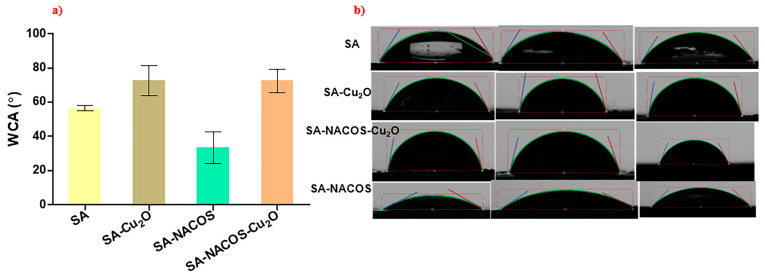
Water contact angle (WCA) testing results for the sodium alginate (SA) film and the different composite (SA-Cu_2_O, SA-NACOS-Cu_2_O, and SA-COS-Cu_2_O) films: (**a**) WCA values; (**b**) image (three replicates) of a water drop on the composite surface for WCA testing.

As illustrated in Figure 6 and Table 2, the pure SA film exhibited a WCA of about 56.6 ± 1.6°, confirming its hydrophilic surface. The inclusion of Cu_2_O (i.e., SA-Cu_2_O) increased the WCA to 72.7 ± 8.8°, suggesting a modest enhancement in hydrophobicity due to the hydrophobic properties of Cu_2_O. In contrast, the SA-NACOS composite exhibited a lower WCA than pure SA, indicating increased hydrophilicity. Notably, the addition of Cu_2_O to the SA-NACOS composite (i.e., SA-NACOS-Cu_2_O) raised the WCA to 72.4 ± 6.8°, a value comparable to that of SA-Cu_2_O. This demonstrates that Cu_2_O predominantly governs the surface hydrophilicity of SA-based composites, irrespective of the presence of NACOS. Due to the inherently high hydrophilicity of chitosan oligosaccharides (COS), measuring their contact angle was not feasible.

### 3.4. Antibacterial Activity

**Figure 7:**
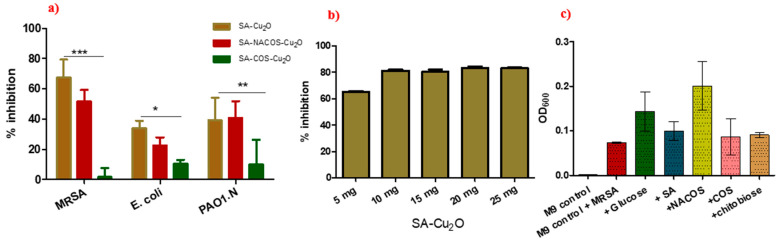
Antimicrobial activity assay results: (**a**) % inhibition calculated for the different composites (SA-Cu_2_O, SA-NACOS-Cu_2_O, and SA-COS-Cu_2_O) against MRSA, *E. coli*, and PAO1. Statistical significance was determined using a two-way ANNOVA. Asterisks indicate significance levels, with *p <* 0.05 (*), *p <* 0.01 (**), and *p <* 0.001 (***), as determined by the statistical test; (**b**) % inhibition against varying concentrations of SA-Cu_2_O (mg·mL^−1^); (**c**) growth rate of MRSA (OD_600_) at different carbon sources, including glucose, SA, NACOS, COS, and chitobiose. Error bars represent standard deviations.

All Cu_2_O-containing composites—SA-Cu_2_O, SA-NACOS-C_2_O, and SA-COS-Cu_2_O—demonstrated inhibitory effects against all the tested bacterial strains (*S. aureus*, *E*. *coli*, and *P*. *aeruginosa*) (see Figure 7a and Table 2). The SA-Cu_2_O composite, devoid of chitin-derived oligosaccharides, exhibited the highest inhibition against the Gram-positive strain *S. aureus*, followed by the Gram-negative strains *E*. *coli* and *P*. *aeruginosa*. These findings align with the previous research by Safaei and Taran (2018) [14], who reported a significant antimicrobial activity of SA-CuO composites against *S. aureus* and *E. coli.*

SA-Cu_2_O demonstrated the highest antibacterial activity against MRSA (67.4 ± 11.9% inhibition), *E. coli* (33.7 ± 5.1% inhibition), and PAO1.N (39.2 ± 15% inhibition). Compared to SA-Cu_2_O, SA-COS-Cu_2_O exhibited significantly reduced activity, with a 97.5% inhibition loss for MRSA (1.7 ± 5.94%, *p <* 0.001, ***), a 69.2% reduction for *E. coli* (10.4 ± 2.7%, *p <* 0.05, *), and a 74.5% reduction for PAO1.N (9.997 ± 16.3%, *p <* 0.01, **). These findings suggest that SA-COS-Cu_2_O is significantly less effective than SA-Cu_2_O against all bacterial strains, particularly *S. aureus* (MRSA). On the other hand, SA-NACOS-Cu_2_O showed antibacterial effectiveness (51.6 ± 7.7% against MRSA, 22.5 ± 5.5% against *E. coli*, and 40.6 ± 11.3% against PAO1.N) comparable to SA-Cu_2_O, with no significant differences (*p >* 0.05).

The percentage of inhibition was assessed at varying concentrations of SA-Cu_2_O, as shown in Figure 7b, with the maximum inhibition observed at 10 mg/mL. No further enhancement in inhibition was detected at higher concentrations.

Furthermore, SA, NACOS, COS, and *N*-*N* diacetylchitobiose [(GlcNAc)_2_] were observed to serve as carbon sources for bacterial growth under minimal conditions, as depicted in Figure 7c. These findings are consistent with the work of Asadpoor et al. [25], who demonstrated that COS could be utilized as a carbon source for the growth of *Staphylococcus*. This contrasts with earlier studies by Abidin et al. [26,27] that reported the antibacterial activity of (GlcNAc)_2_ against *Listeria monocytogenes* and *E. coli*. Notably, chitosan oligosaccharides and *N,N*-diacetylchitobiose did not exhibit significant antibacterial effects, despite chitosan’s well-documented antimicrobial properties under mild acidic conditions, which are attributed to its protonated amine groups. The limited antimicrobial efficacy of these compounds may stem from several factors, including reduced molecular mass hindering effective interaction with microbial cell membranes, the presence of uncharged acetyl groups in *N,N*-diacetylchitobiose diminishing electrostatic interactions with negatively charged microbial surfaces, and the degree of deacetylation influencing the cationic nature of chitosan derivatives. Additionally, antimicrobial sensitivities to chitosan-based compounds vary across species. Intriguingly, the ability of bacteria to utilize diverse carbon sources under minimal conditions suggests a potential metabolic adaptation mechanism, with bacterial self-inhibition possibly occurring upon carbon source depletion.

The above results demonstrate that Cu_2_O serves as the primary antimicrobial agent responsible for bacterial suppression, leading to effective bacterial membrane disruption and reactive oxygen species (ROS) generation. The uniform dispersion of Cu_2_O in the SA matrix, as confirmed by SEM and EDX analyses, likely enhanced the overall antimicrobial activity by maximizing the surface interaction between Cu_2_O and bacterial cells. However, the incorporation of chitin-derived oligosaccharides (especially COS) may attenuate the antimicrobial efficacy of the composites. This reduction arises because these oligosaccharides can function as carbon sources, partially counteracting the antimicrobial effects of Cu_2_O. This underscores the necessity of balancing biofunctionality, potentially conferred by polysaccharides and oligosaccharides, with antimicrobial activity and biodegradability in polysaccharide/inorganic material composites. Interestingly, although all oligosaccharides can effectively support bacterial growth, with NACOS being the most effective (see Figure 7c), only COS exhibited a statistically significant offsetting effect (Figure 7a). The mechanism underlying this phenomenon is worth further investigation.

While enhanced hygroscopicity or surface hydrophilicity of a polymer material generally promotes microbial activity, the observed reduction in the antimicrobial properties of the composites due to chitin-derived oligosaccharides does not correlate with their effects on hygroscopicity or surface hydrophilicity, as previously discussed. This suggests that the variations in antimicrobial activity among the composite samples are predominantly attributed to the distinct capacities of chitin-derived oligosaccharides to function as carbon sources.

Compared to the existing antimicrobial materials used in food packaging and biomedical applications, SA-Cu_2_O composites offer several advantages. Traditional antimicrobial packaging materials often rely on synthetic polymers such as polyethylene and polypropylene embedded with antimicrobial agents like silver nanoparticles, titanium dioxide, or zinc oxide [10]. While these materials provide effective microbial inhibition, their environmental impact, cost, and potential toxicity raise concerns. Biopolymer-based antimicrobial films have emerged as sustainable alternatives [28]. Chitosan, in particular, has been widely used for its inherent antibacterial properties due to its cationic nature, which interacts with bacterial cell membranes. However, its antimicrobial efficacy varies depending on environmental conditions, such as pH and moisture content. In contrast, the Cu_2_O-loaded SA composite provides a stable antimicrobial effect under diverse conditions due to the sustained release of Cu_2_O and its ability to generate ROS that damage bacterial membranes.

In biomedical applications, hydrogels incorporating antimicrobial agents, such as silver, iodine, or antibiotics, have been used for wound healing and infection prevention. While these materials exhibit strong antibacterial properties, concerns over antibiotic resistance and cytotoxicity have spurred interest in metal-based antimicrobial systems like Cu_2_O [29]. Compared to nanosilver-containing hydrogels, Cu_2_O-loaded SA composites present a cost-effective and biocompatible alternative with comparable broad-spectrum antimicrobial activity. Moreover, the hydrophilic nature of SA allows for controlled swelling and moisture retention, making these materials suitable for wound dressings and tissue-engineering applications.

Despite their advantages, SA-Cu_2_O composites face challenges in optimizing antibacterial performance when oligosaccharides (which are usually biofunctional) are included. The reduction in antimicrobial activity observed in NACOS- and COS-containing formulations suggests that these oligosaccharides serve as additional nutrient sources for bacteria. Future studies should explore strategies such as surface modifications or controlled release mechanisms to mitigate this effect. Additionally, comparative studies evaluating the long-term stability, antimicrobial persistence, and mechanical properties of SA-Cu_2_O composites against commercially available antimicrobial materials will be essential for their practical implementation in the food packaging and biomedical fields.

## 4. Conclusions

This study demonstrates the potential of sodium alginate/cuprous oxide (SA-Cu_2_O) composite materials as effective antimicrobial agents. A structural analysis using UV-vis, FTIR, and EDX confirmed the successful incorporation of Cu_2_O, with SEM revealing uniform dispersion within the SA matrix. Antibacterial assays showed that all Cu_2_O-containing composites exhibited strong inhibitory effects against both Gram-positive (*S. aureus*) and Gram-negative (*E. coli and P. aeruginosa*) bacteria. Compared to SA-Cu_2_O, the addition of chitin-derived oligosaccharides, particularly COS, significantly reduced the antimicrobial performance, likely due to their function as carbon sources that promoted bacterial growth. However, this reduction was insignificant when NACOS was incorporated. Swelling and water contact angle measurements indicated that the antimicrobial effectiveness of the composites was independent of their hydrophilicity. These findings underscore the importance of carefully designing biopolymer–inorganic antimicrobial composites, as certain matrix components, particularly oligosaccharides, may counteract antibacterial effects.

Future work should prioritize optimizing the composition of these composites to enhance antimicrobial efficacy while minimizing the nutrient-like characteristics of polysaccharide additives. Additionally, comprehensive studies are needed to assess the application-related properties and stability of these materials under real-world conditions, including exposure to temperature fluctuations, humidity, and UV radiation, particularly in food-packaging applications. Moreover, a rigorous evaluation of the environmental and health impacts—encompassing biodegradability, toxicity, and biocompatibility—is essential to ensure the safety and sustainability of these composites.

## Figures and Tables

**Table 1:** Different samples involved in this work, including their codes and compositions.

Code	Composition
SA	SA
SA-Cu_2_O	SA/Cu_2_O = 1:2 (*w*/*w*)
SA-NACOS-Cu_2_O	SA/chitin oligosaccharide/Cu_2_O = 1:1:2 (*w*/*w*)
SA-COS-Cu_2_O	SA/chitosan oligosaccharide/Cu_2_O = 1:1:2 (*w*/*w*)
SA-NACOS	SA/chitin oligosaccharide = 1:1 (*w*/*w*)
SA-COS	SA/chitosan oligosaccharide = 1:1 (*w*/*w*)

**Table 2:** Summary of key properties of the different samples.

Sample	Swelling Ratio (%) at 6 h	Contact Angle (°)	Antibacterial Activity (Inhibition % Against MRSA)
SA	160.4 ± 12.2	56.6 ± 1.6	–
SA-Cu_2_O	18.6 ± 6.0	72.7 ± 8.8	Highest (67.4 ± 11.9%)
SA-NACOS	203.9 ± 17.3	33.3 ± 9.2	–
SA-NACOS-Cu_2_O	90.1 ± 4.9	72.4 ± 6.8	High (51.6 ± 7.7%)
SA-COS	116.1 ± 10.3	N/A ^a^	–
SA-COS-Cu_2_O	16.1 ± 3.9	N/A ^a^	Low (10 ± 16.3%)

Note: ^a^ Contact angle could not be measured due to the samples being too hydrophilic.

## Data Availability

The data underlying this study are available at https://doi.org/10.15125/BATH-01469.

## Abbreviations

The following abbreviations are used in this manuscript:

COS	chitosan oligosaccharide
NACOS	chitin oligosaccharides
SA	sodium alginate
WCA	water contact angle
